# Guided Internet-Based Cognitive Behavioral Therapy in Japanese Patients With Obsessive-Compulsive Disorder: Protocol for a Randomized Controlled Trial

**DOI:** 10.2196/18216

**Published:** 2020-06-24

**Authors:** Kazuki Matsumoto, Sayo Hamatani, Takuya Makino, Taku Uemura, Futoshi Suzuki, Seina Shinno, Tomoki Ikai, Hiroyuki Hayashi, Chihiro Sutoh, Eiji Shimizu

**Affiliations:** 1 Research Center for Child Mental Development Chiba University Chiba Japan; 2 Japan Society for the Promotion of Science Tokyo Japan; 3 Research Center for Child Mental Development University of Fukui Fukui Japan; 4 Department of Child and Adolescent Psychological Medicine University of Fukui Hospital Fukui Japan; 5 Department of Integrated Medical Sciences Graduate School of Medicine University of Fukui Fukui Japan; 6 Department of Integrated Advanced Medicine Graduate School of Medicine University of Fukui Fukui Japan; 7 Department of Primary Health Care Faculty of Medicine University of Fukui Fukui Japan; 8 Department of Emaergency and General Medicine University of Fukui Hospital Fukui Japan; 9 Department of Cognitive Behavioral Physiology Graduate School of Medicine Chiba University Chiba Japan

**Keywords:** internet-based cognitive behavioral therapy, cognitive behavioral therapy, obsessive-compulsive disorder, randomized controlled trial, protocol

## Abstract

**Background:**

Cognitive behavioral therapy for obsessive-compulsive disorder has been established, but access to this therapy in Japan is limited. Internet-based cognitive behavioral therapy may improve treatment accessibility and sufficiently improve obsessive-compulsive symptoms. There are few randomized controlled trials examining the effectiveness of internet-based cognitive behavioral therapy in patients with obsessive-compulsive disorder. We designed a randomized controlled trial protocol to assess the effectiveness of guided internet-based cognitive behavioral therapy in Japanese patients with obsessive-compulsive disorder.

**Objective:**

We aimed to develop a protocol for a randomized controlled trial of internet-based cognitive behavioral therapy in Japanese patients with obsessive-compulsive disorder.

**Methods:**

The randomized controlled trial will compare internet-based cognitive behavioral therapy treatment and usual care groups, each consisting of 15 participants (n=30) diagnosed with obsessive-compulsive disorder. We will evaluate the effectiveness of a 12-week intervention. The primary outcome of symptom severity will be measured using the Yale-Brown Obsessive-Compulsive Scale. Secondary outcomes will be assessed with the Obsessive-Compulsive Inventory, Beck Anxiety Inventory, Patient Health Questionnaire-9, Generalized Anxiety Disorder-7, Working Alliance Inventory-Short Form, and the Euro Qol – 5 Dimension. All measures will be assessed at weeks 0 (baseline) and 12 (follow-up). In the statistical analysis comparing treatment effects, the least-squares means and their 95% CIs will be estimated by analysis of covariance with the change in total outcomes scores at week 12. All comparisons are planned, and all *P* values will be two-sided, with values <.05 considered statistically significant.

**Results:**

The study will be performed from January 2020 to March 2021, and results are expected to be available in mid-2021.

**Conclusions:**

The trial will demonstrate whether internet-based cognitive behavioral therapy improves access and is more effective than more usual care for patients with obsessive-compulsive disorder in Japan.

**Trial Registration:**

University Hospital Medical Information Network (UMIN) 000039375; https://upload.umin.ac.jp/cgi-open-bin/ctr/ctr_view.cgi?recptno=R000044422

**International Registered Report Identifier (IRRID):**

DERR1-10.2196/18216

## Introduction

### Background

Obsessive-compulsive disorder (OCD) has been defined as a common, chronic, and long-lasting disorder in which a person has uncontrollable, reoccurring thoughts (obsessions) and/or behaviors (compulsions) that he or she feels the urge to repeat [1]. OCD is characterized by uncomfortable and painful obsessions and repeated obsessions. The 12-month prevalence of anxiety disorder including OCD in the Japanese adult general population is 5.3%, making it the most common psychiatric disorder [2]. Systemic reviews and meta-analysis show that cognitive behavioral therapy (CBT) is the most effective treatment for OCD [3] and is recommended as first-line therapy by the treatment guidelines of The National Institute for Health and Care Excellence in the United Kingdom (UK NICE) [4]. Telemedicine or remote treatment for patients living in rural areas via internet-based cognitive behavioral therapy (ICBT) has been established as a standard of care in Stockholm, Sweden [5]. In ICBT, patients and therapists interact primarily via email, with treatment including routine work such as explaining the symptoms of the patient’s condition and introducing coping skills based on cognitive behavioral science. A systematic review with meta-analysis comparing 21 studies of face-to-face and guided self-help CBT (mostly ICBT) in 810 patients showed no clear differences in their treatment effect: The effect size was Cohen *d*=–0.02, and face-to-face CBT was smaller than ICBT [6]. Unlike ICBT for anxiety and depression, effectiveness of ICBT for OCD has been investigated in clinical trials. Japanese patients with OCD have significantly improved symptoms with ICBT via videoconference and the method achieved high acceptance [7,8]. Prior study results suggest guided ICBT with minimal therapist intervention may be as effective and accepted by Japanese patients with OCD as ICBT via videoconference [9].

### Internet-Based Cognitive Behavioral Therapy for Obsessive-Compulsive Disorder

Previous randomized controlled trials for obsessive-compulsive disorder have been performed in Sweden, the United States, and Korea [10-12]. In a previous randomized study of 101 patients with OCD assigned to ICBT and online supportive psychotherapy followed by blinded assessments [10], ICBT showed significant improvements in the intervention group compared to the control group. Obsessive-compulsive symptoms as measured by the Yale-Brown Obsessive-Compulsive Scale (Y-BOCS) were improved, and the effect size was high (Cohen *d*=1.12). Another study of 56 patients with OCD included ICBT, reading therapy, and waiting groups [11]. The results of that study suggested that ICBT has a large effect size (Cohen *d*=1.57) among the waiting group at pre- and post- treatment. In Asia, a Korean research team conducted a randomized controlled trial and reported that the treatment group showed significantly improved symptoms over the waiting group, and 25.9% (17/42) responded to treatment, with a within-group effect size of 1.64 (Cohen *d*) [12]. Thus, ICBT, like face-to-face CBT [13], has been shown to be highly effective in the treatment of OCD.

More clinical trials are needed to draw general conclusions regarding the efficacy of ICBT because of substantial country-to-country variations in cultural background, the spread of information, availability of communication equipment, and literacy. Prior research has been performed in countries where the environment is conducive to ICBT, especially as developed countries have a well-established computerized social infrastructure [14]. The penetration rate of information and communication equipment in Japan is more than 90% of all households [15], and there is a favorable social infrastructure for verifying the effectiveness of ICBT. In Japan, our research team confirmed that all patients were in remission by conducting CBT on 3 patients with OCD in their 20s and 40s in the ICBT case series [9]. Based on this achievement, we developed an ICBT program that includes an electronic learning (e-learning) system and chat app. From January 2020 to March 2021, we are conducting a randomized controlled clinical trial in patients diagnosed with OCD.

### Objective

This paper describes the study protocol for a randomized controlled trial designed to evaluate the clinical effectiveness of ICBT versus usual care (UC) among patients diagnosed with OCD.

## Methods

### Study design

**Figure 1:**
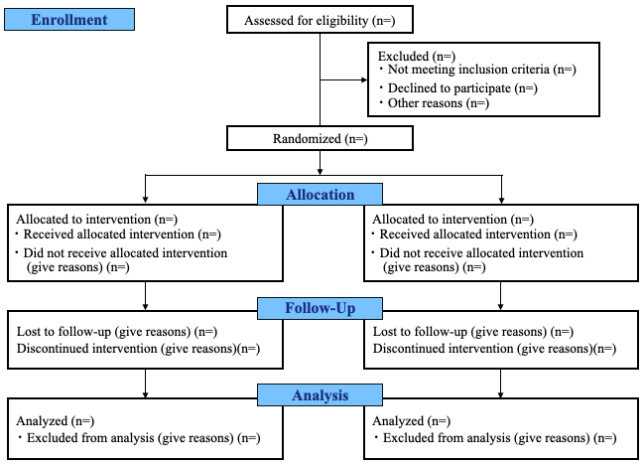
CONSORT flow diagram of a parallel randomized trial with two groups.

This study was designed as a prospective blinded randomized trial with two parallel intervention groups consisting of a 12-week treatment regime of UC alone or ICBT combined with UC (Figure 1) [15-18].

#### Participants and Eligibility Criteria

Inclusion criteria for this study include individuals aged 15-60 years having a primary diagnosis of OCD according to the Mini-International Neuropsychiatric Interview (MINI) and remaining symptomatic [19,20] with symptoms rated at least moderate in severity, based on a Y-BOCS score >14 [21,22], and with sufficient skills to send email and access the e-learning system. Participants with psychosis or organic mental disorder, or with a current high risk of suicide, substance abuse or dependence within the 12 months prior to enrollment, antisocial personality disorder, or unstable medical conditions will be excluded.

#### Recruitment

The planned recruitment rate is 3 participants per month from January 2020 to December 2020, or until a total of 30 participants are recruited. Participants will be recruited by placing informational posters and leaflets at medical institutions in Chiba and Fukui Prefecture and on the institutional homepage. All participants will continue to be treated by their general practitioners, whose permission must be granted prior to study enrollment. This study will be conducted at the outpatient clinic of Chiba University Hospital and Fukui University Hospital in Japan.

### Interventions

#### Guided ICBT program

The ICBT program participants will engage in one program learning session per week. After working on the ICBT, the patient will email the therapist about their thoughts and questions about the study and behaviors suggested by the program for them to try in daily life. The therapist, as general rule, will contact the patient within 24 hours to encourage the patient's efforts, raise questions, and advise on CBT techniques to increase the effectiveness of treatment. The ICBT program consists of 12 weekly lessons, including the following elements: ICBT program guidance, psychological education and case conceptualization of obsessive-compulsive disorder, setting treatment goals and creating an anxiety hierarchy, explaining the behavioral experiment of catastrophic interpretation and the exposure-response disturbance method, and preventing recurrence.

#### Software

We will use LearningBox, a system developed by Tatsuno Information Systems [23] and MediLine, a medical chat service developed by Shar Medical to provide ICBT [24]. LearningBox (Multimedia Appendix 1) is an e-learning system that allows administrators to easily create and manage educational materials, manage members, and save and view grades. Video and PDF teaching materials can also be posted on the site and distributed to specific users. The e-learning system can store and manage user results, but personal information is not stored during the present trial.

MediLine (Multimedia Appendix 2) is a medical chat service (medical social networking service) that replaces email and phone calls. It has strong encryption against military-level information leaks. MediLine communicates in a double-encrypted state according to the Japanese government guidelines [25-28]. Encryption is performed end-to-end in real time in the server, during communication, and in temporary memory at the terminal. In other words, it is designed so that information cannot be extracted during data transfer. Since identifiers are issued by in-house introduction, hijacking and spoofing are prevented. The staff registered by the organization can delete the account at the time of retirement and cannot view it thereafter, so there is no provision for information to be taken out of the hospital after retirement [24].

#### Usual Care (Control group)

Participants will be permitted to continue using antidepressant or other medicines during the study period. Participants' primary doctor will have right to change medication, to refer participants for counselling, and to secondary care if deemed clinically appropriate. All changes in conventional treatment, along with the reasons for those changes, will be recorded. Participants in the control group will be offered the ICBT treatment after the trial if they did not improve under the control conditions.

### Outcomes

#### Baseline and Clinical Characteristics

Baseline characteristics will include gender, age, educational attainment, marital status, employment status, age at onset and duration of OCD, medications, and intelligence as measured by the Japanese Adult Rating Test (JART) [29,30].

#### Primary Outcome

The primary outcome will be measured by the Y-BOCS, which is a rated questionnaire consisting of 10 questions across two subscales, Obsession and Compulsion [21,22].

#### Secondary Outcomes

Secondary outcomes will include health-related quality of life, symptoms of depression and generalized anxiety, and therapeutic relationship. We will measure health-related quality of life with the EuroQol - 5 Dimension (EQ-5D) [31,32], the psychological bond between therapist and participant using the Working Alliance Inventory-Short Form (WAI-SF) [33], depressive symptoms using the Patient Health Questionnaire-9 (PHQ-9) [34], generalized anxiety symptoms using the Generalized Anxiety Disorder-7 (GAD-7) [35], and the Beck Anxiety Inventory (BAI) [36].

### Sample Size

We used the statistical analysis software G*power 3.1 to estimate sample size of an unmatched t-test [37]. Sample size was estimated as 14 participants per group. The effect size predicted in this study is at least 1.00 from the two previous two clinical trials [8-10], the directionality of the test is a two-sided test, the significance level was set at 0.05%, the test was two-sided, and the test power (1-β) was set to 80%. The study will require a minimum 14 participants per group so we are setting a target enrollment of 30 participants to allow for a 5% dropout rate.

### Randomization

Eligible participants will be randomly assigned to the ICBT or UC group at a ratio of 1:1, with assignments made using the minimization method, ensuring a balance in baseline Y-BOCS scores (Y-BOCS ≥20) and gender.

### Statistical Analysis Plan

Statistical analysis and reporting will be conducted in accordance with CONSORT (Consolidated Standards of Reporting Trials) guidelines [38], with primary analyses based on the intention-to-treat principle. For baseline variables, summary statistics will be constructed, employing frequencies and proportions for categorical data and mean and SD for continuous variables. Baseline variables will be compared using the Fisher exact test for categorical outcomes and the unpaired *t*-test for continuous variables. For the primary analysis comparing treatment effects, the least-squares means and their 95% CIs will be estimated by analysis of covariance (ANCOVA) with the change in total Y-BOCS scores at week 12. This ANCOVA model will account for the variation caused by treatment effects, and gender and baseline Y-BOCS scores will be entered as covariates. Analyses of secondary outcomes will be performed in the same manner. All comparisons are planned, and all *P* values will be two-sided. *P* values <.05 will be considered statistically significant. All statistical analyses will be performed using the latest version of SAS (SAS Institute Inc).

### Ethics and Dissemination

This study will be conducted at the Academic Outpatient Clinics of Chiba University and Fukui University in Japan. When potential participants contact the study trial office through the Chiba University, they will be informed of the study objectives and be asked if they are willing to participate by phone screening. Each participant will then be required to provide written informed consent for their participation in this study. Each participant will be informed that all participants will receive UC from their general practitioner and that half of the recruited participants will also receive ICBT in addition to their UC. All adverse events will be reported, and serious adverse events will be immediately reported to the Institutional Review Board of Chiba University Hospital in addition to being registered with the hospital risk management system. An adverse event will be defined as a symptom or disease occurring during the clinical trial, whether related to the ICBT program or not. The study will be conducted and reported according to CONSORT-ETHICS guideline recommendations [39].

This clinical trial protocol was approved by the clinical trial review committee of Chiba University Hospital on November 18, 2019 (G2019017) and approved by the ethics review committee of the Fukui University Graduate School (20190075). It is registered in the University Hospital Medical Information Network database of clinical trials in Japan (UMIN: 000044422).

## Results

The study period is from January 6, 2020 to March 31, 2021. The case registration period is scheduled for 12 months from January 6, 2020 to December 31, 2020.

## Discussion

This randomized controlled study will evaluate the effectiveness of ICBT for patients with OCD. The findings of this study will provide valuable evidence to facilitate development and establish treatment algorithms in ICBT for patients with OCD. The study will also introduce this method for providing ICBT in Japan, as patients will be given access to self-help materials using an e-learning system (LearningBox) and medical chat app (MediLine). The UC control group will be randomly assigned, and the multicenter study design will reduce bias and improve the likelihood of obtaining generalizable results. The study will also have limitations, including the inability to elucidate the specific effects of ICBT program because there is no placebo group to control for nonspecific factors. The study also will not control for medical therapies or treatment resistance. It is unclear whether pharmacotherapy plus CBT improves OCD symptoms more than pharmacotherapy alone [4,40]. Therefore, combination therapy with pharmaceuticals and ICBT should be tested in future trials to determine whether each is beneficial or if therapy is improved by combined therapies.

## Appendix

Multimedia Appendix 1 LearningBox E-Learning System.

Multimedia Appendix 2 The MediLine Chat App.

## Abbreviations

BAI: Beak Anxiety Inventory
CBT: Cognitive Behavioral Therapy
EQ-5D: EuroQol-5-Divison
GAD-7: Generalized Anxiety Disorder-7
ICBT: Internet-Based Cognitive Behavioral Therapy
JART: Japanese Adult Rating Test
OCD: Obsessive-Compulsive Disorder
PHQ-9: Patient Health Questionnaire-9
RCT: Randomized Controlled Trial
UC: Usual care
WAI-SF: Working Alliance Inventory-Short From
Y-BOCS: Yale-Brown Obsessive-Compulsive Scale
