# Supplementary figures and images for: Guided Internet-Based Cognitive Behavioral Therapy in Japanese Patients With Obsessive-Compulsive Disorder: Protocol for a Randomized Controlled Trial
Source: JMIR Res Protoc. 2020 Jun 24;9(6):e18216. doi: 10.2196/18216 (PMC7381023; doi:10.2196/18216)

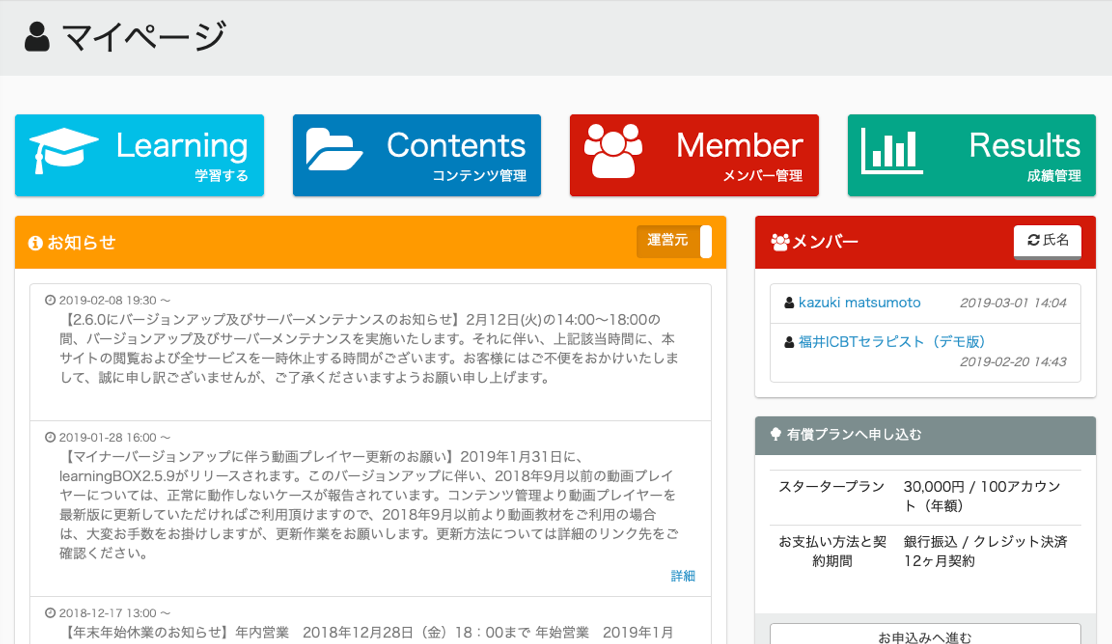

Supplement: Multimedia Appendix 1 [file resprot_v9i6e18216_app1.png]

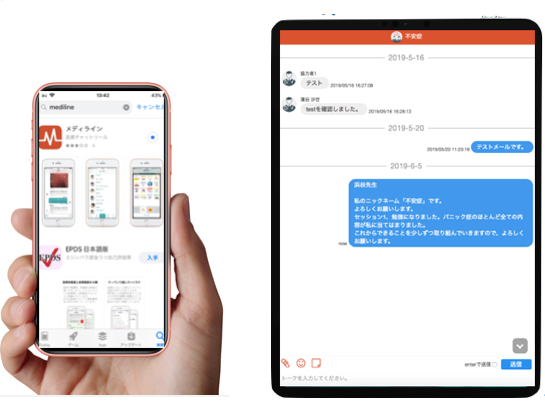

Supplement: Multimedia Appendix 2 [file resprot_v9i6e18216_app2.png]
